# Transfer learning for genotype–phenotype prediction using deep learning models

**DOI:** 10.1186/s12859-022-05036-8

**Published:** 2022-11-29

**Authors:** Muhammad Muneeb, Samuel Feng, Andreas Henschel

**Affiliations:** 1 Department of Electrical Engineering and Computer Science, Khalifa University of Science and Technology, Al Saada St - Zone 1, Abu Dhabi, United Arab Emirates; 2 Department of Science and Engineering, Sorbonne University Abu Dhabi, PO Box 38044, Abu Dhabi, United Arab Emirates

**Keywords:** Bioinformatics, Genotype-phenotype, Transfer learning, Deep learning, Genetics

## Abstract

**Background:**

For some understudied populations, genotype data is minimal for genotype-phenotype prediction. However, we can use the data of some other large populations to learn about the disease-causing SNPs and use that knowledge for the genotype-phenotype prediction of small populations. This manuscript illustrated that transfer learning is applicable for genotype data and genotype-phenotype prediction.

**Results:**

Using HAPGEN2 and PhenotypeSimulator, we generated eight phenotypes for 500 cases/500 controls (CEU, large population) and 100 cases/100 controls (YRI, small populations). We considered 5 (4 phenotypes) and 10 (4 phenotypes) different risk SNPs for each phenotype to evaluate the proposed method. The improved accuracy with transfer learning for eight different phenotypes was between 2 and 14.2 percent. The two-tailed p-value between the classification accuracies for all phenotypes without transfer learning and with transfer learning was 0.0306 for five risk SNPs phenotypes and 0.0478 for ten risk SNPs phenotypes.

**Conclusion:**

The proposed pipeline is used to transfer knowledge for the case/control classification of the small population. In addition, we argue that this method can also be used in the realm of endangered species and personalized medicine. If the large population data is extensive compared to small population data, expect transfer learning results to improve significantly. We show that Transfer learning is capable to create powerful models for genotype-phenotype predictions in large, well-studied populations and fine-tune these models to populations were data is sparse.

## Background

Human characteristics are different for various reasons; classified into nature (genetics and heritability) and nurture (Environment and food consumption). The physical characteristic or the presence of a particular disease is known as a phenotype. For example, eye color is a phenotype, and blue, green, brown, and black eye colors are the four possibilities of this particular phenotype. Genetic data (genotype, DNA) plays a vital role in determining some characteristics or diseases. Researchers inferred that the variations or mutations in the DNA result in the variation in the phenotype. Mutations at a particular location in the DNA that can be used to find this phenotype-genotype relationship are SNPs, known as Single-nucleotide polymorphism. SNPs associated with an increased risk of developing a particular disease or trait are called risk SNPs.

Following is a list of acronyms used in this manuscript:

- PhenotypeSimulator = PS
- Utah Residents with Northern and Western European ancestry = CEU
- Yoruba in Ibadan, Nigera = YRI
- Single-nucleotide polymorphism = SNPs
- Genome-wide association studies = GWAS
- Artificial neural network = ANN
- Recurrent neural network= RNN
- Gated recurrent unit = GRU
- Long short-term memory = LSTM
- Bidirectional LSTM = BILSTM

In genetics, case/control studies for humans, animals, and different species [1–7] are of great importance for the diagnosis of a particular disease in organisms. Genotype data available for genetic analysis can significantly improve the final results [8]. Although next-generation sequencing [9, 10] and imputation algorithms [11, 12] have increased the genetic data available for analysis, genomics is failing on diversity [13]. There are still some small understudied populations [14, 15] for which data is not enough for genetic analysis or case/control classification. We refer to a population as small, if the available genotype data for analysis is insufficient for reliable conclusions [16], i.e., a statistical power analysis would deem the dataset to contain too few samples to achieve statistical significance. To mitigate this issue, we can use knowledge extracted from a large population (for which data is in abundance), to make predictions about the small population with a fine-tuned model [17]. Indeed, transfer learning is currently an increasing research sub-field of machine learning. It is used in computer vision [18], natural language processing tasks like sentiment analysis [19], and to improve the clustering of single-cell RNA-Seq data [20]. Furthermore, it is often used in conjunction with deep neural networks [21], which require a lot of data and computing power.

Pio et al.  [22] used transfer learning to improve the human gene regulatory network reconstruction accuracy using gene-related metabolic features (generated through gene expression data) from human and mouse. The task at hand is to identify regulatory links between genes in a network. This approach is similar to what we present in this work, but instead of using it for gene network reconstruction, we use it for classification of genotype-phenotype prediction tasks.

Mignone et al. [23] also uses a specific variant of transfer learning in which the number of negative examples or the labels for particular class instances are missing (negative samples), but positive samples are known. In that case, positive interactions between genes are known, but negative interaction are not. They derived the information from the common genes between humans and mouse, and used those features to train the machine learning model, and used that model to identify the negative interactions. Similarly, this article [24] used the sample approach to the prediction of virus-human protein-protein interactions.

Transfer learning is also used for genetic data [25–27] and offers several advantages described below.

- Transfer learning helps to solve complex real-world problems having little or almost no labeled data availability [17].
- It allows transferring knowledge from one model to another based on domains and tasks.
- With transfer learning, the final model achieved has better generalization ability than directly training the data on a small population.

In the following, we elaborate on the applications and advantages of using transfer learning, particularly for genotype data.

The motivation for using transfer learning is personalized medicine [28], which is a medical model that separates people into different groups with medical decisions and products being tailored to the individual patient based on their risk of disease. Genotype data for one person belonging to a particular population cannot train the deep learning model. However, with transfer learning, using genotype data of another population, we can make some valuable insights about that person, leading to personalized medicine for a specific person. In the case of humans, cancer is the perfect example. Suppose we have 1000 cancer patients from the CEU (Northern Europeans from Utah) population and 10 cancer patients from the YRI (Yoruba in Ibadan) population. Direct statistical analysis on 10 (YRI) cancer patients would not lend itself to a Genome Wide Association Study, as it would be underpowered for this purpose. However, using genotype data from 1000 (CEU) cancer patients can improve case/control classification. Most deep learning algorithms work best when given a lot of data [29], and we can leverage samples from large populations to make case/control predictions on small populations, after adjusting for the characteristics of the small population. Many species have biological function resemblances [30], so transfer learning using genotype information would be an invaluable resource for the effective management of breeding programs and cases/controls studies in small and endangered populations. Consider Bornean elephants (smallest Asian elephant subspecies) [31] which is among endangered species. Suppose that the Bornean elephants, which are small in quantity, suffer from some disease. In that case, we would not have enough genetic information for any analysis or prediction. However, we can use case/control studies of other elephant species that are large in quantity to get insights into the Bornean elephants.

Deep learning models perform well when the amount of available datasets is sufficiently high.The deep learning models trained on the large population store the knowledge that can be used for classification; however, these models do not contain the exact representation required for classifying small population samples, so we used fine-tunning (transfer learning) to improve the representation learned by the models using the training data from the small population to improve the classification score. Deep learning models store the information in various layers, which can be made trainable/non-trainable. It helps to extract the particular knowledge or update the stored knowledge allowing for fine-tuning the model to improve the classification score.

The second reason is that existing tools like Plink and LDPred-2 consider the linear interaction of SNPs weighted by the corresponding Odds ratio to find the polygenic risk scores. These models cannot be directly used for transferring knowledge as it will result in only SNP transfer learning methodology with limited information from the large population.

In general, deep learning algorithms perform well when the dataset is large, whereas the performance is poor when the dataset is in low quantity. With that in mind, we used classic machine learning algorithms, which are good when the dataset is low. Second, the genotype-phenotype prediction depends on the phenotype under consideration. We first tested classic machine learning and then employed a deep learning algorithm for prediction. So, if classical machine learning algorithms can work for transfer learning, there is no need to use deep learning algorithms.

We considered only simulated data for testing the applicability of transfer learning for genotype data and the following are the reasons for this:

- Phenotype: For transfer learning, data for a specific phenotype for both populations is required, and we did not have the data for a specific phenotype for both populations. If we directly use the 1000 Genome data, we can have a phenotype for all people, but for the large population, data will be low, which is one of the assumptions for successful transfer learning.
- Heritability: Second, the performance of the deep learning models depends on the dataset’s quality and heritability [32]. The real dataset mostly has missing values, which may reduce the number of common SNPs between both populations, whereas simulated data has fewer missing values for SNPs.
- Different populations: If the dataset is a mix, one has to classify people into different populations. Moreover, there are many possibilities for transfer learning. We transferred knowledge from CEU to YRI, whereas transfer from YRI to CEU is also possible. Due to multiple possibilities, we restricted the scope to a particular transfer to test the applicability.

The proposed method is distinct from the existing studies for the following two reasons. We searched for existing transfer learning methodologies for genotype data, but we did not find any article specifically designed for genotype data. Second, the method presented in the article can assist in studying the transfer learning approach for various populations. For example, we considered CEU as a large population and YRI as a small population, but they can be interchanged too. Phenotypes with various heritability and number of risk SNPs can be generated, and the effectiveness of transfer learning can be studied. All the above reasons make this article distinct, and there is no such article tackling the above issue.

Section  (Material, Module 1 to 3) contains the data generation part. Section (Methods, Module 4 to 5) provides technical context and describes the entire pipeline for transfer learning. Section (Results) demonstrates the results. Section (Discussion) contains a closing discussion.

## Materials

This section contains the datasets used in this manuscript. We explained the data generation part in detail for the following reasons.

**Fig. 1:**
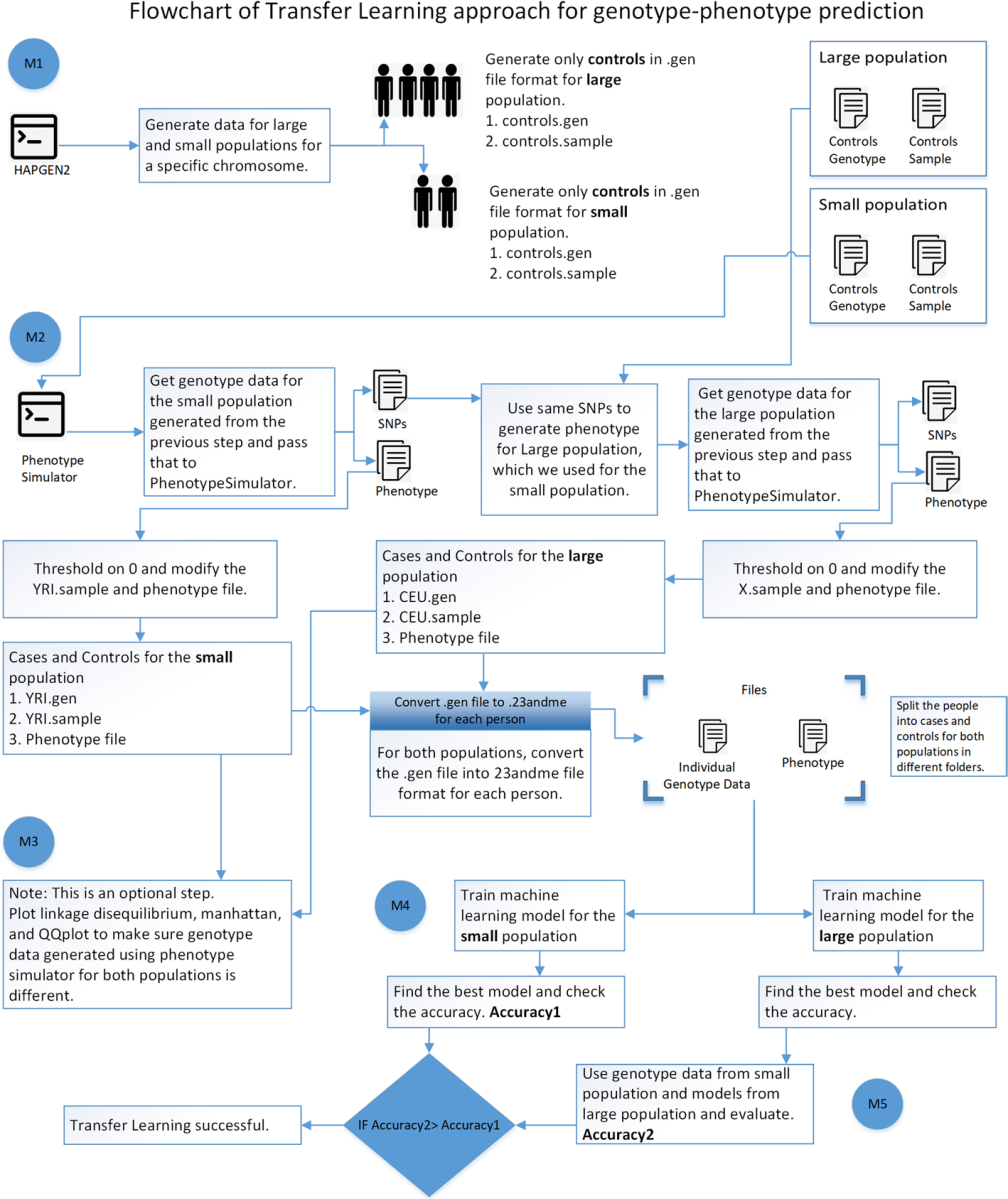
This diagram shows the flowchart of the deep learning-based transfer learning approach for the genotype-phenotype prediction that consists of 5 modules

We worked on simulated data, and using the tools used to generate data, one can generate data with multiple possibilities. For instance, genotype data with varying heritability, genetic variation, and the number of risk SNPs can be generated, and the effectiveness of transfer learning for various phenotypes can be explored. It may work for some simulated phenotypes and may give negative results for others. A dataset with the same parameters must be generated for both populations to support such studies, making the data generation process crucial in the pipeline. The overall methodology is explained in Fig. 1.

The transfer learning approach for the actual genotype data is straightforward, but when working on simulated genotype data, there are various parameters, which can change the outcome of the whole process, and it requires an in-depth explanation of all the processes involved in the data generation part. These processes include handling directory structure and file formats, coordinating genotype data between populations, and applying transfer learning to a small population. As we presented the pipeline along with the use case, the data generation part also includes the information for the use case to comprehend the methodology section. We feel not explaining this section in detail would miss the overall gist of the manuscript, making it possibly difficult to understand.

### Module 1: Generate data using HAPGEN2

We generated genotype data for YRI (Small population) and CEU (Large population) using HAPGEN2, which is a tool that takes 1000 Genomes Pilot + HapMap 3 data [33], and simulates cases/controls genotype data by specifying the disease model through a set of disease-causing SNPs together with their relative risks [34].

- 1000 controls for CEU and 100 controls for YRI
- Generated genotype data for Chromosome 21
- This step generates four files: YRI.gen and YRI.sample (genotype data and sample file for the small population), CEU.gen, and CEU.sample (genotype data and sample file for the large population)

### Module 2: Generate phenotype using phenotype simulator

PhenotypeSimulator takes genotypes to simulate the basic genetic, non-genetic covariates, observational noise, and non-genetic correlation structures. The effect structure of the four abovementioned components is divided into a shared effect across traits or an independent effect for a number of traits, allowing for complex phenotype structures. Finally, the simulated phenotype and its components are saved into genetic output formats. Using PS, generate phenotype for each sample in the YRI.samples and CEU.samples files, and perform further analysis on common SNPs between the two populations. Transfer learning requires training a machine learning model on the common SNPs between both populations because each SNP is a feature when training the model. If SNP is missing from the small population, we cannot transfer knowledge.

- Number of SNPs in CEU: 101053
- Number of SNPs in YRI: 136021
- Number of common SNPs between CEU and YRI: 62283

#### Generate phenotype for the small population

YRI.sample and YRI.gen files, generated in the previous step, are passed to PS to simulate a phenotype for each person. We considered the default values for each parameter as shown on page 3,example 2, https://cran.r-project.org/web/packages/PhenotypeSimulator/vignettes/PhenotypeSimulator.pdf in the PhenotypeSimulator documentation. A detailed explanation of each variable is mentioned in PhenotypeSimulator documentation https://cran.r-project.org/web/packages/PhenotypeSimulator/PhenotypeSimulator.pdf. We generate 4 phenotypes for 5 risk SNPs and 10 risk SNPs, labeled with a specific code as shown below.

- 4 phenotypes with 5 risk SNPs: YRI_5_1, YRI_5_2, YRI_5_3, and YRI_5_4
- 4 phenotypes with 10 risk snps: YRI_10_1, YRI_10_2, YRI_10_3, and YRI_10_4
- The actual risk SNPs are different and randomly decided by PS for each phenotype.

#### Generate phenotype for the large population

CEU.sample and CEU.gen file, generated in the previous step, are passed to PS to simulate the phenotype, using the same parameters and the same risk SNPs to simulate the data for the small population.

- 4 phenotypes with risk SNPs 5: CEU_5_1, CEU_5_2, CEU_5_3, and CEU_5_4
- 4 phenotypes with risk SNPs 10: CEU_10_1, CEU_10_2, CEU_10_3, and CEU_10_4
- One crucial point is that the actual risk SNPs for YRI_5_1 are the same for CEU_5_1, and the machine learning model trained on CEU_5_1 is used for the prediction of YRI_5_1.
- PS also outputs the risk SNPs for the small population, which can be extracted and passed to PS as causal SNPs when generating data for the large population pass.

Each phenotype is represented in this format, X_Y_Z, X is the population code (CEU or YRI), Y is the number of risk SNPs (5 or 10), and Z is the iteration number (1 to 4). The number of risk SNPs (Y) can be mutated in the PS. Z is a specific iteration. We generated a new dataset for each iteration, and the risk SNPs selected by PS are different for the same Y.

At this stage, we have genotype data for eight phenotypes. Considering these two datasets, CEU_5_1 and YRI_5_1, we explained the next steps in the pipeline, and these steps are the same for each phenotype.

#### Convert continuous phenotype to cases/controls

PS generates continuous phenotype, which we converted to binary phenotype after thresholding on 0 (below 0 a control and above 0 is a case), for CEU_5_1 and YRI_5_1. Represent controls with 0 and cases with 1, and update the CEU_5_1/CEU.sample and YRI_5_1/YRI.sample files accordingly with new phenotypes values after thresholding. Generate a separate phenotype file for both populations, which contains the sample id and the phenotype, and convert CEU_5_1/CEU.gen and YRI_5_1/YRI.gen files in 23andme file format, so the machine learning techniques specified in this article [35] are applicable to genotype-phenotype prediction.

### Module 3: Plot linkage disequilibrium, manhattan, and QQplot

**Fig. 2:**
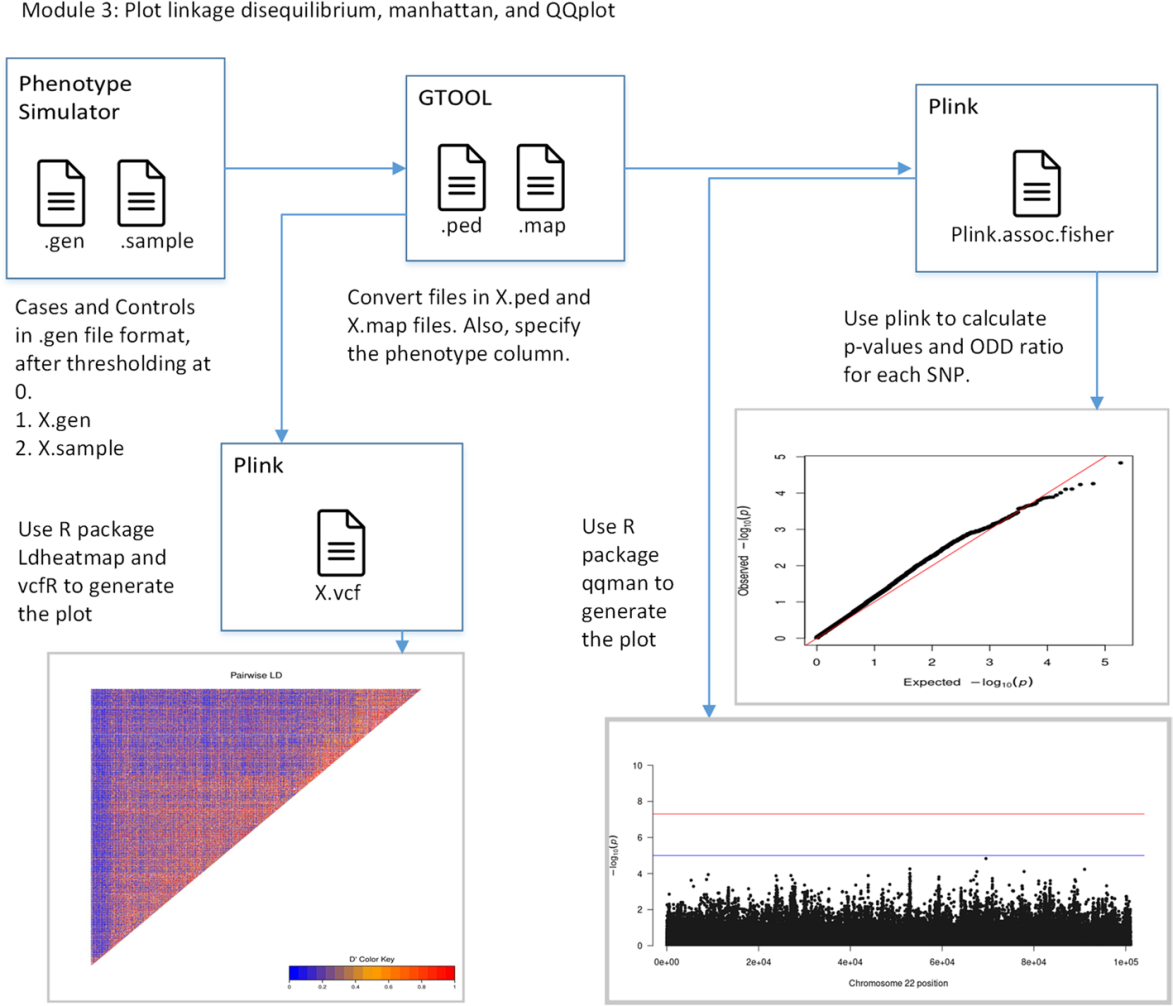
Module 3: Plot linkage disequilibrium, manhattan, and QQplot. Genotype data generated from PS is passed to Gtool -> Plink to convert genotype data into VCF file format, and that file is used to generate linkage disequilibrium. Gtool output is also used to find p-values for each SNP and generate the manhattan plot

Module 3 is optional used to generate the manhattan, QQ, and Linkage disequilibrium plot for a particular phenotype of both populations. Figure 2 elaborates the visualization step. For visualization, use X_5_1/X.gen and X_5_1/X.sample files.

## Methods

This section provides in-depth detail of the proposed pipeline. In each section, some subsections describe the sub-steps in each module.

### Module 4: Machine learning for both small and large populations

At this stage, we have these files for both populations.

- Genotype files in 23andme file format.
- SNPs file
- Phenotype

#### Quality control

Quality control steps are performed on the genotype data to ensure the quality of the data is of a high standard for any interpretation [36–38], but for the simulated data, some quality control steps, like removing SNPs with missing alleles and duplicate SNPs, can be skipped. Perform these steps independently for both large and small populations.

#### SNPs pre-selection

Using 62,283 SNPs for training may overfit the model, so SNPs pre-selection process (*p*-value threshold or mutation difference between cases/controls at each SNP [35]) will reduce the dimensionality of input data leading to a generalized model. Generate multiple datasets using the SNPs pre-selection process on the training data for both populations. Tables 1 and 2 show the number of SNPs in each sub-dataset of the small population and the large population for each phenotype.

**Table 1:** 13 sub-datasets for each phenotype containing different number of SNPs

SNPs in sub-dataset	1	2	3	4	5	6	7	8	9	10	11	12	13
YRI_5_1	3	6	14	32	51	105	154	236	287	435	822	1112	1803
YRI_5_2	1	4	7	26	28	60	65	192	233	314	662	935	1868
YRI_5_3	0	1	10	21	38	67	116	202	239	436	728	995	1616
YRI_5_4	4	5	24	83	127	153	193	255	295	487	732	1048	1729
YRI_10_1	2	7	18	47	91	137	180	345	422	551	1086	1899	
YRI_10_2	7	11	12	67	136	171	248	279	494	563	656	1158	2032
YRI_10_3	3	5	13	43	65	103	130	146	341	431	590	1110	1974
YRI_10_4	1	3	20	36	75	109	179	264	312	561	913	1282	2130

0 means sub-dataset does not contain any phenotype and such sub-datasets are ignored

**Table 2:** 10 sub-datasets for each phenotype containing different number of SNPs

SNPs in sub-dataset	1	2	3	4	5	6	7	8	9	10
CEU_5_1	17	18	48	154	193	1135	1196	3421	4901	6629
CEU_5_2	0	4	18	74	89	949	987	3551	4867	6583
CEU_5_3	0	4	10	73	80	921	1016	3411	4780	6641
CEU_5_4	0	1	28	101	126	932	969	3438	4718	6751
CEU_10_1	2	20	96	101	809	894	3485	4816	6601	
CEU_10_2	1	4	22	150	162	1005	1076	3545	4999	6673
CEU_10_3	1	5	134	243	296	1119	1162	3732	5015	6731
CEU_10_4	0	1	44	151	164	924	980	3522	4962	6699

0 means sub-dataset does not contain any phenotype and such sub-datasets are ignored

#### Machine learning for the small population

Before applying machine learning for genotype-phenotype prediction, we must generate multiple sub-datasets for both populations. Each sub-dataset contains a different number of SNPs. It is essential to try different SNPs to find the model that generalizes well. We generated about 13 sub-datasets containing a different number of SNPs for each phenotype. As the SNPs pre-selection process is performed on the training data, we must extract the same SNPs from the test data. Make sub-datasets like this YRI_5_1/snps_X, where X represents the number of SNPs in the particular sub-dataset. The number of SNPs selected for a specific dataset depends on the linear threshold value. Dataset split for YRI population is like this.

- Training data 70 samples (YRI_TD)
- Validation data 10 samples (YRI_VD)
- Test data 20 samples (YRI_ED)
- Final test data 50 samples (YRI_FD)

For genotype-phenotype prediction of the small population we used five machine learning algorithms: SVM = Support vector machine [39], Cart = Decision tree classifier [40], Rus = Random under sampler, Forest = Random forest classifier [41], Ub = Bagging classifier. We trained each sub dataset on these 5 models and selected the sub-dataset which performed well on the test data. For example, if YRI_5_1/snps_1000/SVM generated the best accuracy for the test data YRI_ED, save that model and test newly generated data YRI_FD.

- To test the generalization of the best model, generate a new dataset YRI_FD.
- A new dataset shows how well genotype-phenotype prediction for the small population works without transfer learning.
- Use this newly generated data for the evaluation of the transfer learning methodology.

Table 3 shows the results of best machine learning without transfer learning for both YRI_ED and YRI_FD datasets.

**Table 3:** The first, second, third, fourth, and fifth columns show the phenotype, the optimal number of SNPs, the best model for the YRI_ED dataset, accuracy on the YRI_ED dataset, and the final accuracy on the YRI_FD dataset

Phenotype	SNPs	Best model	YRI_ED accuracy	YRI_FD accuracy
YRI_5_1	236	UB	66	56
YRI_5_2	26	CART	85	52
YRI_5_3	10	SVM	61	44
YRI_5_4	1048	UB	71	42
YRI_10_1	1086	SVM	76	48
YRI_10_2	11	Random forest	66	52
YRI_10_3	1110	CART	71	52
YRI_10_4	36	SVM	61	46

#### Machine learning for the large population

For each phenotype of the large population, we generated about ten sub-datasets containing a different number of SNPs. For each sub-dataset, we used nine different architectures of deep learning algorithms ANN (3, Table 4 shows the architecture), LSTM(2), GRU(2), and BILSTM(2) (Table 5 shows the architecture) for transfer learning; after training, save all models.

**Table 4:** Model 1, 2, 3 architecture

	Model 1	Model 2	Model 3
Layers	Parameters	Parameters	Parameters
Layer 1—FullyConnected	Input layer	Input layer	Input layer
Layer 2—FullyConnected	30	80	80
Layer 3—FullyConnected	10	70	70
Layer 4—FullyConnected	2	40	50
Layer 5—FullyConnected	–	10	20
Layer 6—FullyConnected	–	2	10
Layer 7- FullyConnected	–		2

The number of layers and the number of neurons in each layer can vary. Moreover, the hyper-parameters can be tuned to improve the final performance. The number of trainable and non-trainable layers can vary, but transfer learning does not perform well if all layers are trainable and the performance is improved

**Table 5:** Model (4, 5, 6), (7, 8, 9) architecture

	Model 4/5/6	Model 7/8/9
Layers	Parameters	Parameters
Layer 1—FullyConnected	Input layer	Input layer
Layer 2—GRU/LSTM/BILSTM	50	50
Layer 3—GRU/LSTM/BILSTM	20	20
Layer 4—FullyConnected	2	10
Layer 5—FullyConnected	–	5
Layer 6—FullyConnected	–	2

The number of layers and the number of neurons in each layer can vary. Moreover, the hyper-parameters can be tuned to improve the final performance. The number of trainable and non-trainable layers can vary, but transfer learning does not perform well if all layers are trainable and the performance is improved

Following is the dataset split for training the machine learning model on the CEU population.

- Training data 700 samples (CEU_TD)
- Validation data 100 samples (CEU_VD)
- Test data 200 samples (CEU_ED)

**Fig. 3:**
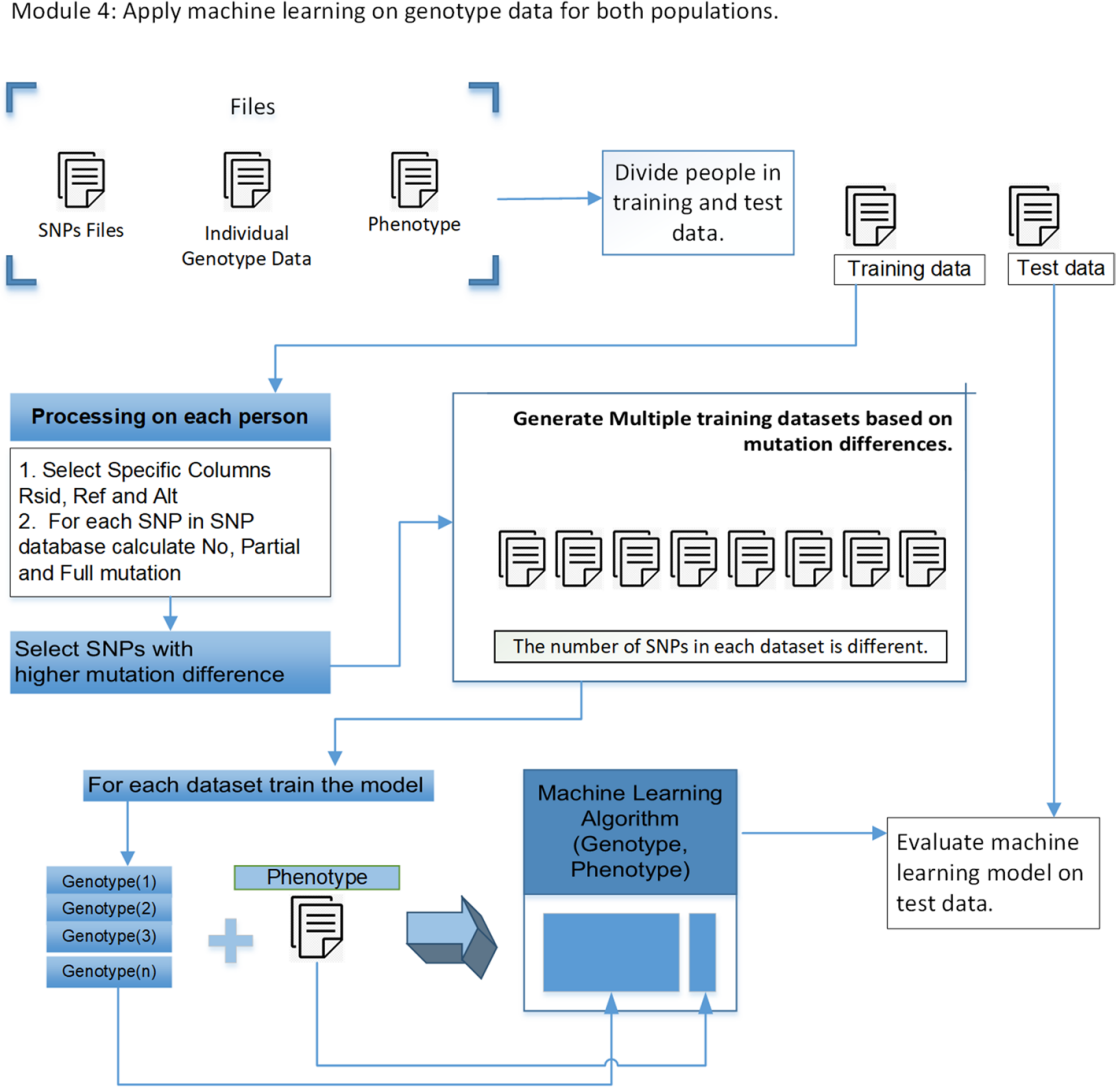
Module 4: Machine learning approach for training the large population genotype data. Train the machine learning model for a large population, and save all models. We can also choose those models for which the test accuracy is high, but we would have a limited number of models to test when evaluating the transfer learning performance

Figure 3 shows the machine learning methodology for genotype-phenotype prediction.

### Module 5: Transfer learning for the small population

**Fig. 4:**
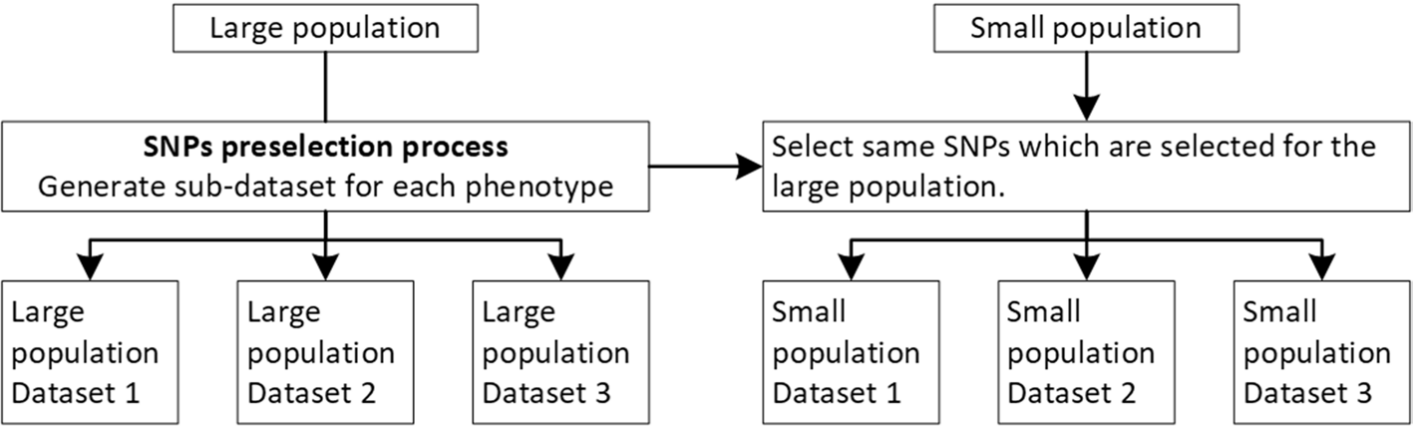
The SNPs selection process for the small population to use transfer learning for a particular phenotype. Before the SNPs preselection process, it is necessary to select the common SNPs between both populations using Rsid

In the case of transfer learning, we have to select SNPs from two populations. If the machine learning model trains on N features (encoded SNPs) from the large population (CEU_5_1/snps_1000/), then we have to choose the same SNPs from a small population (YRI_5_1/snps_transfer_1000/). For each sub-dataset of the large population, make a corresponding sub-dataset of the small population, which contains the same SNPs in the large population as shown in Fig. 4.

In two ways, transfer learning methods can be used for genotype data.

#### Transfer learning with SNPs transfer learning

SNPs selected to classify large population datasets into cases/controls based on mutation differences are good features for machine learning. Same SNPs can also be used for the classification of a small population. For each sub-dataset for a particular phenotype train the 5 classific machine learning algorithm on (CEU_5_1/snps_transfer_1000/training data), to find the optimal sub-dataset test the model on (YRI_5_1/snps_transfer_1000/test data) and for generalization test the model on (YRI_5_1/snps_transfer_1000/YRI_FD).

#### Transfer learning with deep transfer learning

**Fig. 5:**
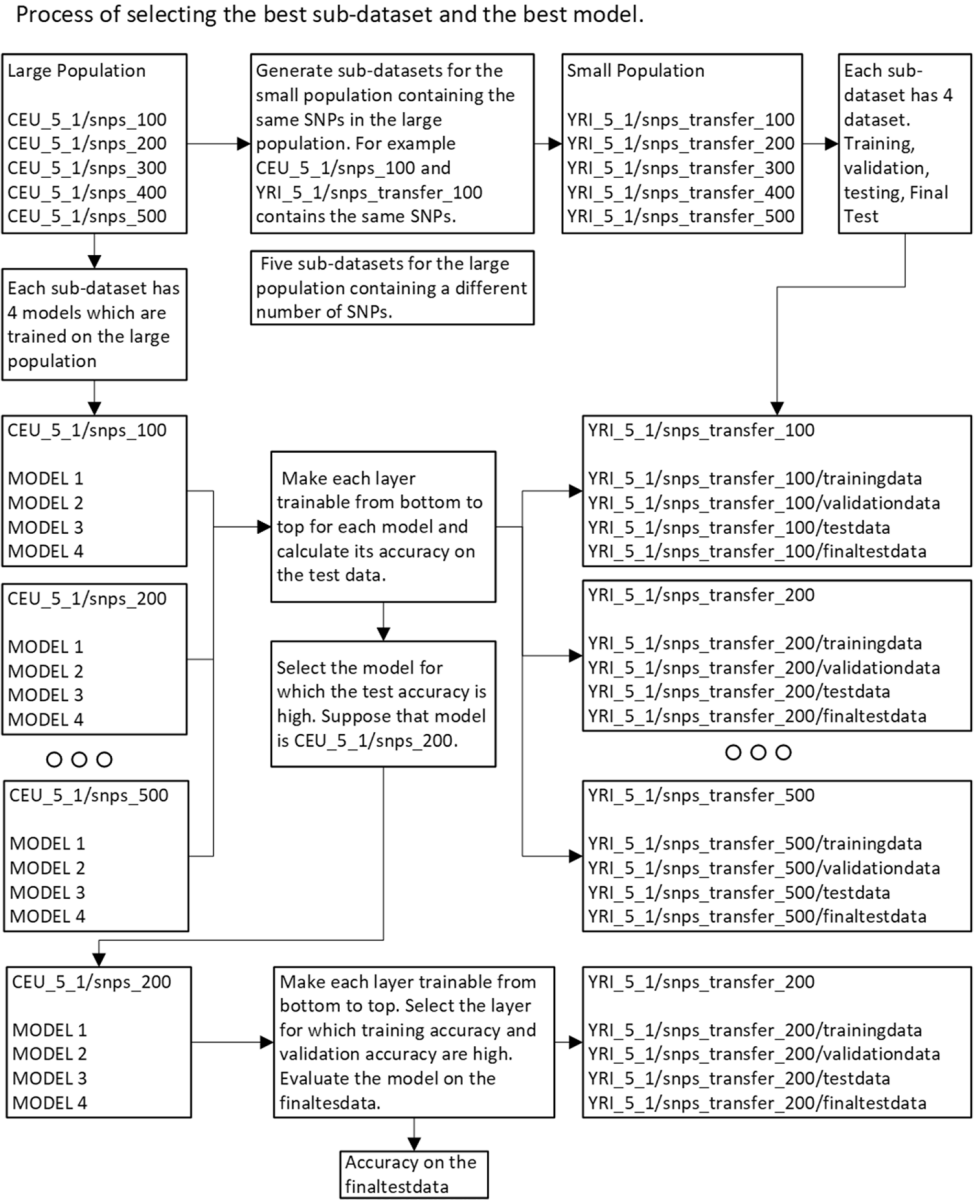
The process of selecting the best sub-dataset and model for transfer learning. Choose the model for which the training, validation, and test accuracy is high and test the final model on the final dataset

In deep transfer learning, use the model trained on the large population for the small population genotype-phenotype prediction. Models trained on the large population can substantially increase the classification accuracy of the small population. The procedure to use deep transfer learning is explained in the following bullets and graphically shown in Fig. 5.

- For each large population sub-dataset (CEU_5_1/snps_X, X is the number of SNPs in sub-dataset), train 9 deep learning models.
- For each large population sub-dataset (CEU_5_1/snps_X), generate a corresponding sub-dataset for the small population containing the same SNPs of the large population (YRI_5_1/snps_transfer_X).
- For each model (CEU_5_1/snps_1000_Model1), make layers trainable and nontrainable from bottom to top. When the model is trainable, pass (YRI_5_1/snps_transfer_1000_TD) for training, (YRI_5_1/snps_transfer_1000_VD) for validation, and evaluate the model on (YRI_5_1/snps_transfer_1000_ED). Select models which generate the best accuracy for (YRI_5_1/snps_transfer_1000_ED).
- Repeat this process for each sub-dataset of a particular phenotype.
- There are about 10 sub-datasets for the large population and 9 models for each sub-dataset. Based on the (YRI_5_1/snps_transfer_1000_ED) select the model for the final evaluation on (YRI_5_1/snps_transfer_1000/YRI_FD).

### Models and implementation

This section contains information about the deep neural networks and transfer learning types used in the methodology.

#### Models

For any deep learning model, the number of layers, the number of neurons, and hyper-parameter selection can affect transfer learning performance [42]. We used nine different architectures of deep learning algorithms ANN(3), LSTM(2), GRU(2), and BILSTM(2) for transfer learning. All the models should be trained again if we change the base dataset.

#### Artificial neural network

An ANN goes through a training process where it learns to identify patterns in the input data [43, 44]. It contrasts its actual output generated with what it was intended to achieve during the back-propagation process. The activation function produces non-linearity, which is the most significant benefit of using ANN. Every sub-dataset has a different number of SNPs, so we used an ANN with different processing units in the first layer for each sub-dataset.

#### Recurrent neural network

The recurrent neural networks, known for their memory, which allow them to use past inputs to influence the current input and output, are used for time-series or sequential data such as language translation [45], natural language processing, voice recognition [46] and image captioning and classification [47].

We considered three types of recurrent neural networks: GRU, LSTM, and Bi-directional LSTM.

GRU: This deep learning algorithm consists of two gates (update and reset gate) to predict the next output in the sequence. The update gate determines the amount of prior knowledge to be transferred to the subsequent state, enables the model to copy all prior knowledge if necessary, and eliminates the danger of vanishing gradient. How much of the prior data will be ignored is determined by the reset gate.

LSTM: This deep learning algorithm consists of three gates (input, forget, and output gate) to predict the next output in the sequence. The input gate chooses what data from the input signal and short-term memory from the previous phase should be kept in long-term memory. The forget determines which long-term memory details should be retained or ignored. The output gate creates new short-term memory and transfers it to the cell in the following step using the current input, the old short-term memory, and the freshly computed long-term memory.

BILSTM: In a bidirectional LSTM, we consider two separate sequences (One from right to left and the other in the reverse order) and two LSTM networks.

These algorithms have already been used for genotype-phenotype [48–50] prediction making them applicate for genotype-phenotype transfer learning.

The use of the algorithm depends on the application. For example, CNN and its variants (ImageNet, ResNet, VGG) are good when transfer learning is used for image data. Similarly, models like word2vec and GloVe are used for natural language-based transfer learning. For genotype data, there is no existing model which can be used, so we must design such models from scratch. Among deep learning models, the best algorithms which can be used for transfer learning are ANN and RNN.

### Types of transfer learning

There are three different types of transfer learning we consider here, simple transfer learning, pre-trained models, and fine-tuning.

**Fig. 6:**
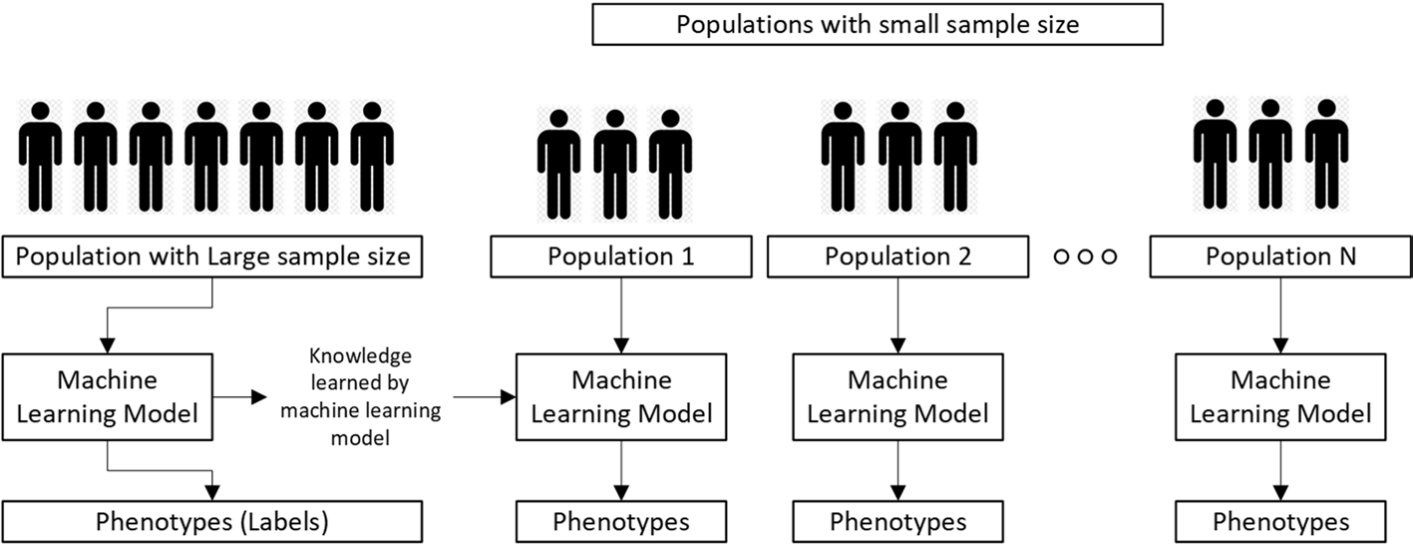
Basic transfer learning mechanism for genotype data. The model trained on the large population can be used to predict multiple small populations, and one has to ensure that before starting the analysis, common SNPs should be selected among all populations

Figure 6 shows the primary mechanism of transfer learning to transfer knowledge for genotype-phenotype prediction.

#### Simple transfer learning

Transfer learning means when the model trained on some task A is used to make predictions for some other but related task B. Similarly, a deep learning model trained on a large population can predict a small population for a particular phenotype. For simple transfer learning, train the deep learning model on the large population, evaluate model performance on test data of the large population, and use that model to predict the small population.

The word “transfer learning” is described using domains and tasks. A domain *D* consists of: a feature space *X* and a marginal probability distribution P(X), where ${ X=\{x_{1},\ldots ,x_{n}\}\in { {X}}}$. Given a specific domain, ${ { {D}}=\{{ {X}},P(X)\}}$, a task consists of two components: a label space *Y* and an objective predictive function ${ f:{ {X}}\rightarrow { {Y}}}$. The function *f* is used to predict the corresponding label *f*(*x*) of a new instance *x*. This task, denoted by ${ { {T}}=\{{ {Y}},f(x)\}}$, is learned from the training data consisting of pairs ${ \{x_{i},y_{i}\}}$, where $x_{i}\in X$ and ${ y_{i}\in { {Y}}}$

Given a source domain ${ { {D}}_{S}}$ and learning task ${ { {T}}_{S}}$ , a target domain ${ { {D}}_{T}}$ and learning task ${ { {T}}_{T}}$ , where ${ { {D}}_{S}\ne { {D}}_{T}}$ , or ${ { {T}}_{S}\ne { {T}}_{T}}$ , transfer learning aims to help improve the learning of the target predictive function ${ f_{T}(\cdot )}$ in ${ { {T}}_{T}}$ using the knowledge in ${ { {D}}_{S}}$ and ${ { {T}}_{S}}$ .

#### Pre-trained model

**Fig. 7:**
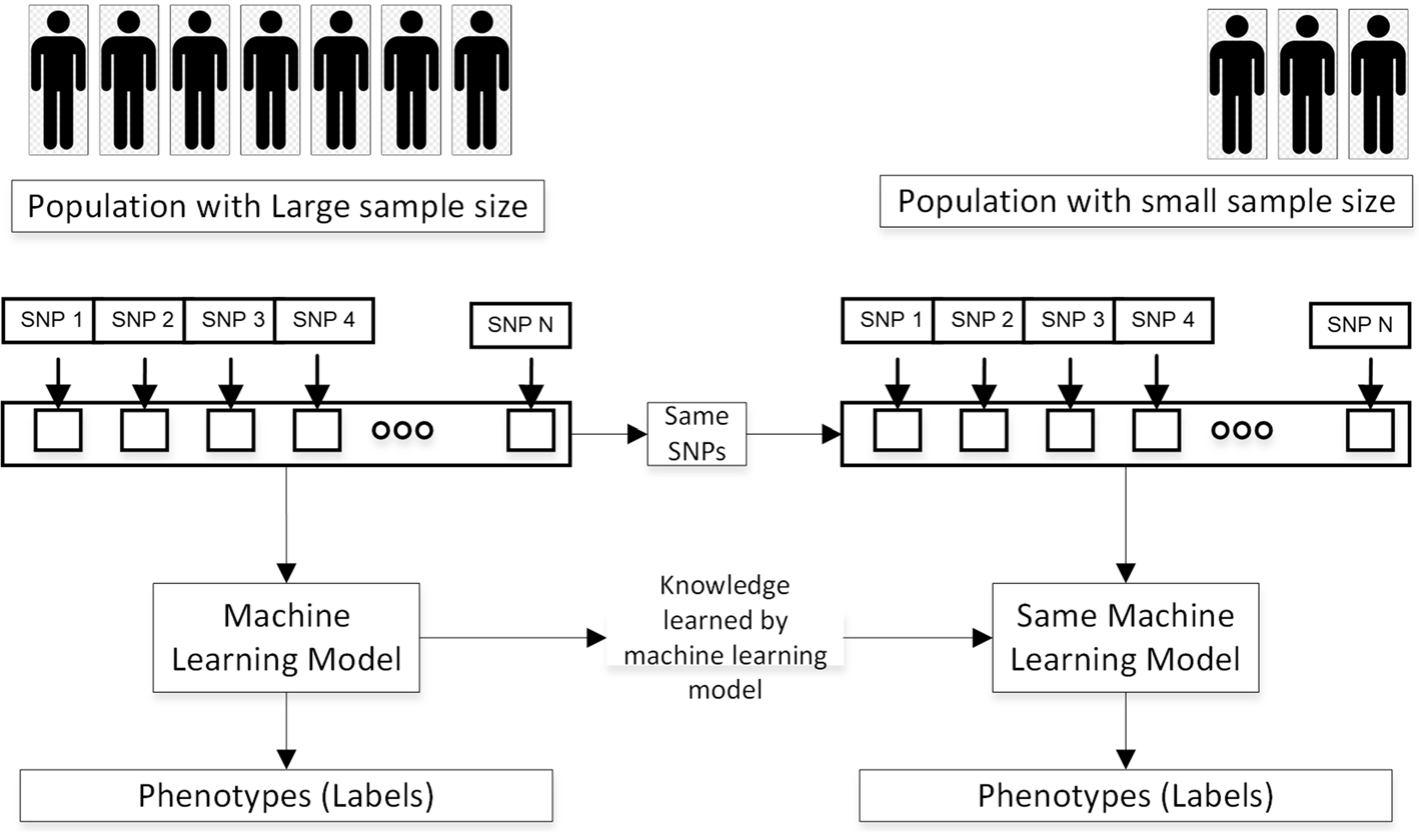
Module 5.2: Transfer learning using pre-trained model. All layers are non-trainable, and the same SNPs (on which the large population model is trained) are passed to the machine learning model for small population classification

Pre-trained type means when a model that has already been learned is used for transfer learning, and the nature of the problem specifies the number of layers to reuse and retrain. For transfer learning, estimation, function extraction, and fine-tuning, Keras contains nine pre-trained models. Many research institutions make trained models accessible regularly. Unfortunately, there is no existing pre-trained model for genotype-phenotype prediction. But it is important to discuss this strategy here. Figure 7 shows the transfer learning mechanism using the pre-trained model for genotype-phenotype prediction.

One important point to understand here is that when we transfer knowledge which features should be used for a small population. From small population data, we will choose the same SNPs for which the initial model was trained using the large population data. In a real dataset, there is a possibility that in a small population, SNPs are missing. In that case, the missing SNPs should be imputed [12] or ignored.

#### Fine-tuning

**Fig. 8:**
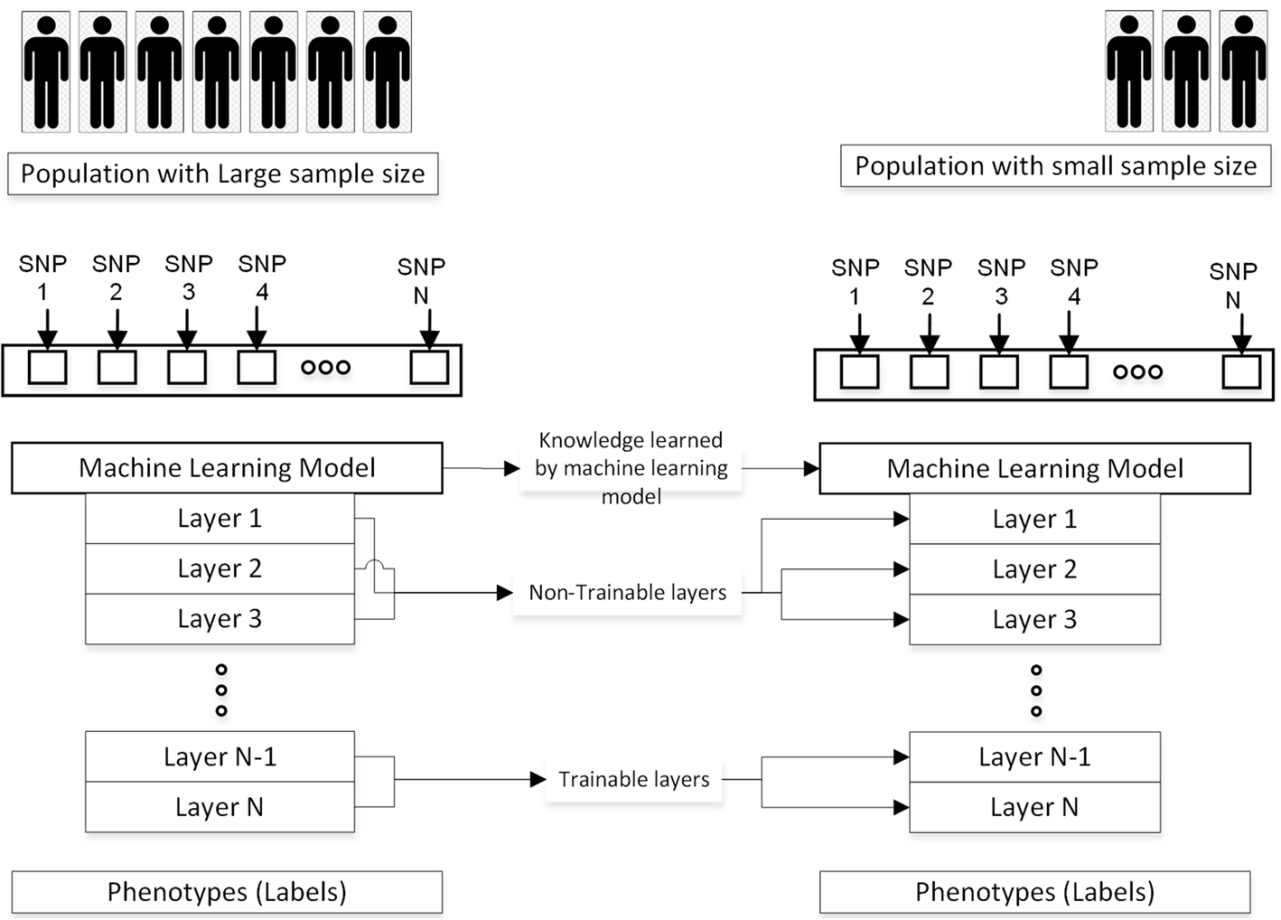
Module 5.3: Transfer learning with fine-tuning. Some of the layers are trainable, and some are not. A different number of trainable and non-trainable layers can be tried to improve the performance of the model

In fine-tuning-based transfer learning, train a deep learning model on the large population, retrain the model on the training data of the small population by making some layers trainable and non-trainable, and finally, evaluate the model on test data of the small population. This technique is common in computer vision because it decreases the size of the dataset required to train the model, which saves time and makes it more suitable for traditional algorithms. Figure 8 shows the transfer learning mechanism using fine-tuning for genotype-phenotype prediction.

## Results

It is important to notice that we should also look for bad results. As we increase the number of SNPs, the deep learning models will be more biased towards the large population, and that is why sub-datasets containing a different number of SNPs and models with different hyper-parameters were generated to generalize the final results [51]. Before using deep learning, it is also important to understand what we will transfer as knowledge. There are two important parameters that we can learn from the large population.

- Selected SNPs from a large population
- Selected SNPs and weights learned by deep learning model from a large population.

### Model 1

When we choose SNPs based on mutation differences to classify large population datasets (B1) into cases/controls, those SNPs can act as good features for machine learning. Same SNPs can also be used to classify a small population (T1). Table 6 shows the results of SNPs transfer learning using a classical machine learning model. Results show that this technique did not work on YRI_FD of each phenotype.

**Table 6:** Results of SNPs transfer learning

Phenotype	SNPs	Best model	YRI_ED accuracy	YRI_FD accuracy
YRI_5_1	236	RUS	66	48
YRI_5_2	26	Random forest	85	54
YRI_5_3	10	Random forest	61	44
YRI_5_4	1048	SVM	71	38
YRI_10_1	1086	SVM	76	46
YRI_10_2	11	Random forest	66	48
YRI_10_3	1110	Random forest	71	48
YRI_10_4	36	RUS	61	32

The first, second, third, fourth, and fifth columns show the phenotype, the optimal number of SNPs, the best model for the YRI_ED dataset, accuracy on the YRI_ED dataset, and the final accuracy on the YRI_FD dataset

### Model 2

We used about 9 different architectures of 4 models ANN, LSTM, GRU, and BILSTM. Results show there is a bit of improvement in the results. Tables 7, 8, and 9 show the results when selected SNPs and weights learned by the deep learning model from a large population are used for transfer learning.

It is worth mentioning here what each layer is learning. In the deep learning model, the top layers correspond to minor features, whereas lower layers are population-specific. The reason that transfer learning may work in the real application is the low genetic diversity in the small populations [52]. So features learned from the large population are more robust than features from a small population. We do not know in advance which layers should be trainable and non-trainable. So, for in-depth analysis, we should try to make each layer non-trainable for each model and then test the model performance. There is also a possibility that the model performs negatively, which is called negative transfer [17].

**Table 7:** Results of deep transfer learning

Phenotype	SNPs	Best model	Best model accuracy on YRI_ED
CEU_5_1	1196, 1196, 4901	Model 1,2,5	66
CEU_5_2	3551, 6583	Model 3,5	75
CEU_5_3	921, 80	Model 2,4	71
CEU_5_4	126,932,932,969	Model 2,3,4,5	71
CEU_10_1	3485, 6601	Model 4,6	71
CEU_10_2	150,150,162,3545	Model 6,9,4,2	76
CEU_10_3	134, 134, 5015	Model 5,7,7	76
CEU_10_4	164	Model 4	76

The first, second, third, and fourth columns show the phenotype, the optimal number of SNPs, the best model for the YRI_ED dataset, and the accuracy of the YRI_ED dataset. These models are further used on YRI_FD

**Table 8:** Results of deep transfer learning for 5 risk SNPs

	5_1	5_2	5_3	5_4
Results without transfer learning	56	52	44	42
With snps transfer learning	48	54	44	38
Improvement with snps transfer learning	− 8	2	0	− 4
With deep transfer learning	58	58	64	56
Improvement with deep transfer learning	2	6	20	14
Max achieved	58	58	64	56
Final improvement	2	6	20	14

The two-tailed *P* value equals 0.0306, t = 2.8146, df = 6, standard error of difference = 3.731

**Table 9:** Results of deep transfer learning for 10 risk SNPs

	10_1	10_2	10_3	10_4
Results without transfer learning	48	52	52	46
With snps transfer learning	46	48	48	32
Improvement with snps transfer learning	− 2	− 4	− 4	− 14
With deep transfer learning	62	66	54	52
Improvement with deep transfer learning	14	14	2	6
Max achieved	62	66	54	52
Final improvement	14	14	2	6

The two-tailed *P* value equals 0.0478, t = 2.4803, df = 6, standard error of difference = 3.629

### Computational analysis

Computation time depends on several factors like the number of neurons in each layer $(l_1, l_2, ... l_N)$, the number of layers in a model *N*, model type (ANN or LSTM), the number of epochs *E*, the number of trainable layers for transfer learning *t*, and the number of neurons in each trainable layer for transfer learning $(l_N,l_{N-1},..l_t)$, where $t>1$.

Consider the time to train the neurons in layer *L* for one iteration is $T_l$, where *l* ranges from $(1 - N)$.

Among the three types of transfer learning, we used only two, and each transfer learning type has a different computation time. Consider the computation time for the following two cases: Transfer learning with SNPs transfer learning and Transfer learning with deep transfer learning.

*Transfer learning with SNPs transfer learning*. This process involves extracting the SNPs from the large population using the p-value threshold on the large population’s GWAS summary statistic file *O*(1), extracting the values for the corresponding SNPs from the small population’s genotype data *O*(1), and training/testing the machine learning model on the small population genotype data $O(E*(T_1+T_2+..T_N))$. The total computation time is $O(1)+O(1)+ O(E*(T_1+T_2+..T_N))$, and it was approximately 10 minutes for one dataset and one machine learning model.

*Transfer learning with deep transfer learning*. The time to train the model on a large population’s genotype is $O(E*(T_1+T_2+..T_N))$. When transferring knowledge from a large population, one must decide the number of trainable and non-trainable layers. If the number of trainable layers is = 0, the final computation time would be $O(E*(T_1+T_2+..T_N))$. If some layers are trainable *t*, the actual computation time would be $O(E*(T_1+T_2+..T_N)) + O(E*(T_N+T_{N-1}+..T_t))$, where is *t* is the number of trainable layers from bottom to top. It was approximately 20 minutes for L = 5, t = 2, and E = 50.

## Conclusion

Machine learning and transfer learning already exist in the literature, but we applied these methodologies for genotype data to show that transfer learning is applicable for genotype data. The whole pipeline requires an in-depth explanation of all the processes involved, like handling directory structure and file formats [53], coordinating genotype data between populations, and applying transfer learning to a small population. As highlighted in the manuscript, we explained these processes and developed sub-modules for processing data. For instance, when generating data for both populations, how to produce symmetric between both datasets.

Any algorithm, TCA, CORAL, 1DCNN, and SVC can also be used for transfer learning, and there is a possibility that these algorithms yield more accuracy when transferring knowledge. So, in the model section, any number of algorithms can be employed without affecting the methodology. We worked on simulated data, and when transferring knowledge to the real dataset for a specific phenotype (cancer or type-2 diabetes), results would be clearer and more interpretable.

Other parameters can also be used, for example, sensitivity, specificity, F1 score, and AUC. The dataset we considered is almost balanced, so accuracy and AUC are almost the same in this case. The other point is that when the best model among multiple models is to be selected for the final testing, we have to decide based on validation accuracy or AUC, so there is a possibility that the model which gives the best AUC is different from the one which gave the best accuracy. It is recommended to select one evaluation metric when evaluating the final performance of transfer learning.

We can use this pipeline to analyze endangered species. Although we tested this approach on a generated dataset, this approach will be more transparent with the real data. However, if the base data is extensive compared to the target data, we can expect transfer learning results to be better.

## Data Availability

Dataset for both populations is available on this link https://mathgen.stats.ox.ac.uk/impute/data_download_1000G_pilot_plus_hapmap3.html.
